# Diseases of the Lungs and Pleuræ

**Published:** 1895-04-20

**Authors:** 


### DISEASES OF THE LUNGS AND PLEURÆ.

### PNEUMONIA.

**Pathology.**—Surgeon-Major Duncan[1], in a paper on Infectious Pneumonia, brought forward some interesting data in connection with an epidemic of pneumonia at Umbala, coming under his observation about ten years ago, in which it appeared that the cases were epidemic and infectious in nature, and not merely local inflammatory. He considered that the idea that cold especially predisposed to pneumonia was conclusively proved to be erroneous by Dr. Sanders, of America (Am. Journal of Med. Sc., July, 1882), who showed that pneumonia, other things being equal, increases uniformly in frequency the nearer we approach the tropics.

**Treatment.**—*Serum.*—Hughes and Carter[2] report fourteen cases of typical pneumonia treated by the hypodermic injection of serum obtained by venesection from convalescent patients. The usual dose was 25 c.c. Only five of the cases showed any effect that could be attributed to immune serum, the injection on the fourth day (three cases) and on the fifth day (two cases) being followed by crises and convalescence; but as in four cases the serum was not considered perfect, being taken from a case of abortive pneumonia, the cases may, perhaps, be said to have given satisfactory results in 50 per cent. The authors warn us against using serum taken from patients with kidney lesions, as they have found that the injection of serum drawn from patients with Bright's disease produces nephritis in dogs.

Percy Kidd advises the giving of strychnine hypodermically, one-sixteenth of a grain being dissolved in four or five minims of water and injected subcutaneously, or into the gluteal muscles. This remedy, he considers, to be specially indicated if the tension of the pulse begins to sink, or if the frequency of the beats be much increased, and more especially if the frequency of the respiratory movements also become much increased. He repeats the dose every two hours until three or four doses have been given, then once or twice in the twenty-four hours. In reference to opium, he uses it when severe pain is present which cannot be controlled by other measures, and for insomnia, restlessness, and delirium; but it is contra-indicated when symptoms arise which point to impending exhaustion of the respiratory centre, viz., shallow, laboured respiration, drowsiness, and a tendency to cyanosis.

**Chloride of Calcium** was first used by Surgeon Lieut.-Colonel Crombie (Pract. Apr., 1893), and more recently Moir[4] has given his results in two cases. The first case took over one ounce of the salt in seven days, and the second case, seven drachms in five days. Dr. Moir does not claim that the early crisis in one was brought about by the chloride of calcium, but he considers that the symptoms were favourably modified; while in the second it seemed undoubtedly to keep the temperature more regular and steady, and to conduce to a favourable termination in a patient who had been a hard drinker and was bordering on delirium tremens. He had been treated first for four days with phenacetin and antifebrin, but without much effect.

**Antimony.**—Dr. D. J. Leech,[5] in discussing the relative value of various drugs in pneumonia, refers to the important place formerly accorded to tartar emetic, and concludes, from a comparison of the results obtained under its administration with the more modern methods, that injurious results followed the excessive and routine employment of antimony as well as of venesection, and that some of the deaths recorded as due to pneumonia must be attributed to the drug given to cure it. Against the harmfulness of tartar emetic in pneumonia, which some are inclined to assume, Dr. Leech quotes the unintentional testimony of one of the leading opponents of unduly active treatment, Dr. Hughes Bennett, who treated 129 consecutive cases (1848—65) with only four deaths. Of the 125 which recovered 95 took antimony. Nearly a third had it in doses of $\frac{1}{14}$ to 1 grain, and 7 in doses of $\frac{1}{16}$ to $\frac{1}{6}$ of a grain. The others were treated with acetate of ammonia and $\frac{1}{32}$ gr. of antimony; but it does not appear that any of the four fatal cases took antimony. The treatment of pneumonia by antimony is certainly worthy of reconsideration.

**Cold Applications.**—Dr. W. F. Jackson,[6] of Brockville, who has already published his results in twenty-five cases, with only one death (in the person of a wretched alcoholic woman, who led a miserable life, and who had albuminuria), now publishes his conclusions as to the value of cold applications, based on seventeen additional cases treated by these means. All recovered in a most satisfactory manner, and, with one exception, very promptly. One case was a very severe double pneumonia, complicated with extensive fibrinous pleurisy, but a good recovery was eventually attained, In the course of these series he treated two other cases, but by the prejudices of members of the families he was not permitted to carry out the cold applications. They were, therefore, treated by methods ordinarily in vogue—viz., stimulating applications to the chest, and the administration of drugs to relieve the pain and fever, and the use of stimulants. Both of these cases died.

The ice-water applications were made by means of large towels, or of suitable pieces of cloth, which, having been wrung out of ice water, were tucked about the chest, covered with a piece of flannel, and kept snug with a roller bandage. These were changed as frequently as they approached the body-heat, until the temperature fell to normal. After this they were renewed during another twenty-four hours only as they became dry, after which their use was discontinued. In some cases various heart tonics were admin'stered, as required.

Dr. Jackson concludes that his two series of cases—forty-two in all—are a sufficient test to place the results beyond peradventure, and he claims that these are better results than are ordinarily attained, and utters a word of warning, the result of twenty years' personal experience, against opiates, coal-tar derivatives, and medicinal depressants, which he considers exceedingly dangerous in pneumonia. Having been misled for many years by the theory that the treatment of pneumonia by cold was too dangerous an experiment, and sure to end in disaster, he has been able to completely refute such a view, and is firmly convinced that both physician and patient will be in a happier position when the value of this method is more widely known.

Another advocate of the treatment of acute pneumonia by means of ice-cold applications is Dr. T. J. Mays, who recently brought his experiences before the notice of the Philad. County Med. Soc. This author believes that much of the ill-success that has followed the use of cold in pneumonia is attributable to the fact that it had been often employed as recommended by Jürgensen with a view to reduce the pyrexia by means of cold baths. Dr. Mays holds that the local trouble in the lung is mainly responsible for the fever, and that this constitutes the vulnerable point in the disease, and therefore makes his cold applications directly over the inflamed lung as well as to the head. If the fever and a great deal of the constitutional disturbance of pneumonia depended on the inflammatory process in the lung, then an abatement of the pulmonary disorder would strike at the very root of the difficulty, and it is clear that the measure which accomplished this must be applied continuously and persistently, and not as in typhoid fever at stated intervals. He therefore advises that the affected area be surrounded with ice contained in bags which are wrapped in towels. If the disease is confined to the front base on one side, one good sized ice bag will suffice, but if the exudation extends to the side and back, then at least one more must be applied, and if the disease is more extensive, as many as are required to cover the whole affected area should be put on. As to the length of time for which cold should be used, that must generally be decided by the amount of fever present. Thus, if the fever falls to or near the normal and shows a tendency to remain there, then the ice might be gradually removed, though it is best not to be in too much haste in withdrawing the cold, for frequently before this is off very long the temperature will suddenly rise again. If this takes place, and the temperature remains high after the ice has been reapplied for some time, it is a possible indication that the inflammation has invaded a new field, and is not active in an old one.

The treatment of pneumonia by ice-cold applications and by cold baths has been urged by several practitioners of note during the last few years, but it is so diametrically opposed to those methods which have held the field for many years, that it is hardly to be wondered at that it is viewed with suspicion by most of those medical men who have had no personal experience of its efficacy, or at least of its harmlessness. Dr. Leech[8] refers to the fact that Priessnitz is said to have used cold compresses, and Compagno, of Naples, used cold baths before 1837 in treating pneumonia. Dr. D. B. Lees has treated sixteen cases of pneumonia by ice bags applied to the chest, and Drs. Goodhart, Sansom, and Coupland have had favourable experiences with these methods of treatment. Dr. Mays collected altogether fifty-four cases (including those of Dr. Lees) treated by the application of ice, of which only two were fatal, and Dr. Leech himself concludes that "such records are necessary preliminaries to more exact knowledge, and though they are not yet sufficient to prove that in the treatment of pneumonia, in the adult at least, a method for successfully dealing with pneumonia has been arrived at, they give incentives for further trial, and they certainly seem to show that cold baths and applications are not to be feared, even though, at times, somewhat alarming symptoms arise from their employment. With regard to children, it seems fairly well established that the application of cold to the chest in severe cases is beneficial. Proof of this is found both in the statistics and in the general concurrence of those who have tried it."

[1] Lancet, Jan. 12, 1895. [2] Therap. Gaz., June, 1894; Quart. Med. Journ., Oct., 1894 [3] Pract., 1894, No. 315, p. 183. [4] Pract., Nov., 1894. [5] Med. Chron., Oct.. 1894. [6] Ther. Gaz , Nov. 15, 1894. [7] New York Med. Journ., Nov. 7, 1894. [8] Med. Chron., Oct., 1894.

